# Utility of clinical and MR imaging parameters for prediction and monitoring of response to capecitabine and temozolomide (CAPTEM) therapy in patients with liver metastases of neuroendocrine tumors

**DOI:** 10.2478/raon-2024-0024

**Published:** 2024-04-14

**Authors:** Maria Ingenerf, Christoph Auernhammer, Roberto Lorbeer, Michael Winkelmann, Shiwa Mansournia, Nabeel Mansour, Nina Hesse, Kathrin Heinrich, Jens Ricke, Frank Berger, Christine Schmid-Tannwald

**Affiliations:** Department of Radiology, University Hospital, LMU Munich, Germany; ENETS Centre of Excellence, Interdisciplinary Center of Neuroendocrine Tumours of the GastroEnteroPancreatic System at the University Hospital of Munich (GEPNET-KUM), University Hospital of Munich, Munich, Germany; Department of Internal Medicine 4, University Hospital, LMU Munich, Munich, Germany; Department of Medicine III, University Hospital, University of Munich, Munich, Germany

**Keywords:** neuroendocrine tumors, liver metastases, CAPTEM therapy, clinical parameters, MR imaging, treatment response

## Abstract

**Background:**

This study explores the predictive and monitoring capabilities of clinical and multiparametric MR parameters in assessing capecitabine and temozolomide (CAPTEM) therapy response in patients with neuroendocrine tumors (NET).

**Patients and methods:**

This retrospective study (n = 44) assessed CAPTEM therapy response in neuroendocrine liver metastases (NELM) patients. Among 33 monitored patients, as a subgroup of the overall study cohort, pretherapeutic and follow-up MRI data (size, apparent diffusion coefficient [ADC] values, and signal intensities), along with clinical parameters (chromogranin A [CgA] and Ki-67%), were analyzed. Progression-free survival (PFS) served as the reference. Responders were defined as those with PFS ≥ 6 months.

**Results:**

Most patients were male (75%) and had G2 tumors (76%) with a pancreatic origin (84%). Median PFS was 5.7 months; Overall Survival (OS) was 25 months. Non-responders (NR) had higher Ki-67 in primary tumors (16.5 *vs*. 10%, p = 0.01) and increased hepatic burden (20% *vs*. 5%, p = 0.007). NR showed elevated CgA post-treatment, while responders (R) exhibited a mild decrease. ADC changes differed significantly between groups, with NR having decreased ADCmin (−23%) and liver-adjusted ADCmean/ADCmean liver (−16%), compared to R’s increases of ADCmin (50%) and ADCmean/ADCmean liver (30%). Receiver operating characteristic (ROC) analysis identified the highest area under the curve (AUC) (0.76) for a single parameter for ∆ ADC mean/liver ADCmean, with a cut-off of < 6.9 (76% sensitivity, 75% specificity). Combining ∆ Size NELM and ∆ ADCmin achieved the best balance (88% sensitivity, 60% specificity) outperforming ∆ Size NELM alone (69% sensitivity, 65% specificity). Kaplan-Meier analysis indicated significantly longer PFS for ∆ ADCmean/ADCmean liver < 6.9 (p = 0.024) and ∆ Size NELM > 0% + ∆ ADCmin < −2.9% (p = 0.021).

**Conclusions:**

Survival analysis emphasizes the need for adapted response criteria, involving combined evaluation of CgA, ADC values, and tumor size for monitoring CAPTEM response in hepatic metastasized NETs.

## Introduction

Neuroendocrine tumors (NETs) encompass a diverse group of neoplasms originating from neuroendocrine cells, with a predilection for the gastrointestinal (GI) tract, pancreas, and pulmonary system.^1^ Their indolent progression often leads to delayed diagnosis, rendering curative surgical resection unfeasible.

Among the therapeutic options for metastatic or progressive cases, Capecitabine and Temozolomide (CAPTEM) chemotherapy has emerged as an effective and safe systemic regimen, particularly benefiting patients with well-differentiated pancreatic NETs.^2,3^ Response rates range widely from 17% to 70%, and progression-free survival (PFS) spans 4 to 38.5 months.^1,4,5,6^ Previous investigations into clinical biomarkers like O6-methylguanine DNA methyltransferase (MGMT) expression, alternative lengthening of telomeres (ALT) activation, and Ki-67 index have yielded conflicting results.^1,7^ Thus, the imperative arises for predictive biomarkers to mitigate treatment failures and needless exposure to toxicity.^1^ As such, there is a growing interest in evaluating imaging parameters for prognostic and monitoring purposes in oncologic therapies.

In addition to morphological changes like tumor size, MRI has the capability to display structural and functional data such as diffusion-weighted imaging (DWI). Incorporating both morphological and functional data, multiparametric MRI could offer a more comprehensive insight into subtle shifts in tumor behavior, especially in small growing tumors such as NET. Parameters such as signal intensity (SI) on T1-weighted or T2-weighted images, tumor vascularization, and apparent diffusion coefficient (ADC) derived from DWI are increasingly scrutinized for their predictive and monitoring potential across various therapy regimens.^8,9,10,11^ Notably, no prior study has assessed the utility of these MRI parameters for monitoring therapy or predicting CAPTEM response in patients with hepatic metastasized NETs. Therefore, this study aims to evaluate clinical, morphological, and functional imaging factors for their ability to predict and monitor therapy response in metastatic NET patients undergoing CAPTEM treatment.

## Patients and methods

### Patients

**FIGURE 1.**
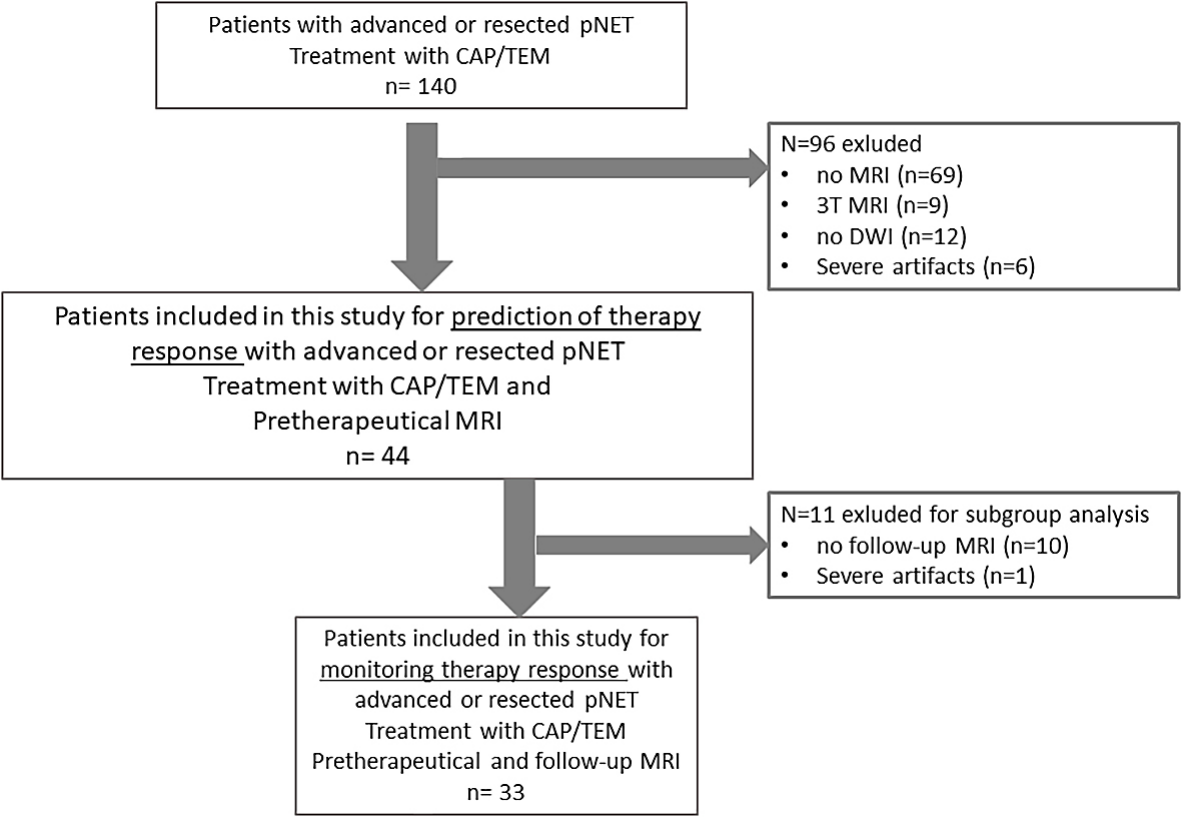
Flow-chart of including process of patients. CAP/TEM = capecitabine and temozolomide; DWI = diffusion-weighted imaging

This retrospective study received approval from the local research ethics committee with decision Number 23-0183 and the requirement for written informed patient consent was waived. We consecutively enrolled patients with histologically confirmed, resected or advanced NETs with liver metastases, all of whom received CAPTEM therapy and underwent pretherapeutic MRI at our department. Furthermore, in the sub-analysis focused on therapy monitoring, we incorporated all individuals from this cohort who underwent subsequent MRI examinations (Figure 1). The timeframe for therapy initiation ranged from April 2013 to June 2022. The decision to commence CAPTEM therapy was reached through consensus in an interdisciplinary tumor conference certified for NETs (ENETS Center of Excellence) for each patient.

### MR imaging

All patients were positioned supine in a 1.5 T MR system (Siemens Healthcare, Erlangen, Germany). For signal reception a phased-array coil was utilized. Images were acquired in accordance with our standard liver imaging protocol. The following sequences were employed for evaluation:

1. A single shot T2-weighted sequence (HASTE).
2. T1-weighted 3D GRE sequences with fat suppression (VIBE) prior to and at 20, 50, and 120 seconds (dependent on circulation time) post intravenous contrast injection (EOB- Bayer Pharma, Germany; 25 µmol/kg body weight).
3. Diffusion-weighted sequences with b-values of 50 and 800 s/mm^2^.
4. After a 15-minute delay, a fat-suppressed T1-weighted VIBE 3D GRE sequence identical to the earlier one.

All sequences utilized parallel imaging with an acceleration factor of 2. ADC maps were computed from the acquired DWI-MR images, incorporating all b-values.

**FIGURE 2.**
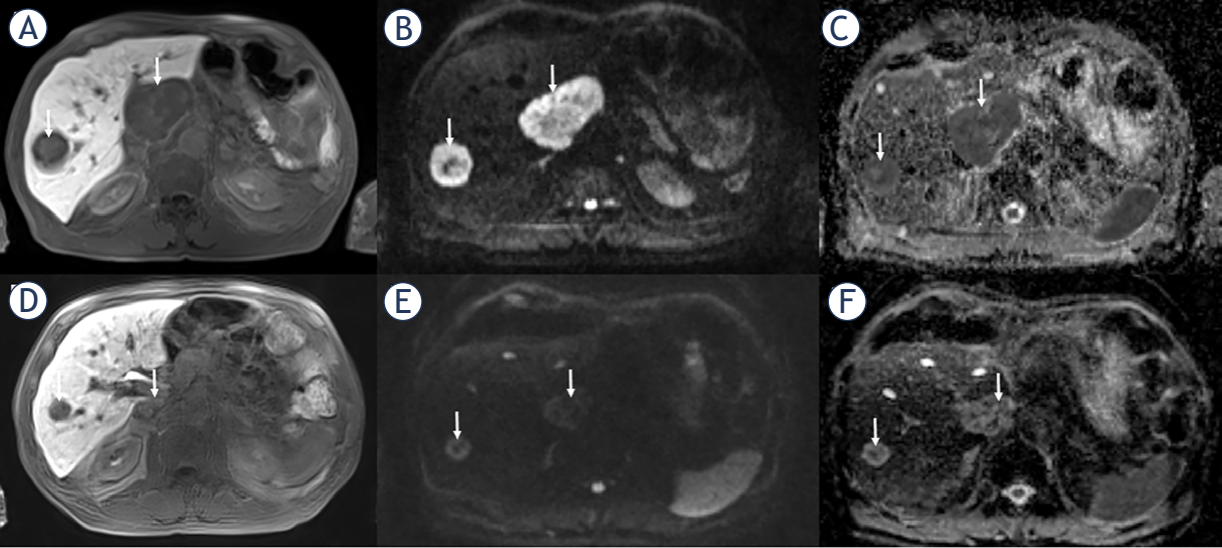
A 72-year-old man with liver metastasis of pancreatic NET classified as responder with a PFS of 38 months. The baseline axial contrast-enhanced T1- weighted image (hepatobiliary phase) **(A)** shows hypointense lesions (arrows) in segment 8 and exophytic in segment 1. The metastases show **(B)** restricted diffusion (arrows) with high signal on axial DW-MR image b = 800 s/mm^2^ and dark signal (arrows) on ADC map **(C)**. After initiation of CAPTEM, the metastases (arrows) exhibited a decrease in size **(D)** On the axial DW-MR image b = 800 s/mm^2^, the metastasis (arrow) **(E)** demonstrated less hyperintense signal to liver and predominantly hyperintense signal (circle) on the ADC map **(F)** indicating less restricted diffusion compared to the preinterventional image. ADC = apparent diffusion coefficient; CAPTEM = capecitabine and temozolomide; DW-MR = diffusion-weighted magnetic resonance; NET = neuroendocrine tumor; PFS = progression-free survival; PR =partial remission; TARE = transarterial radioembolization

### Image analysis

Two board-certified radiologists, blinded to the patients’ clinical and follow-up data, reviewed all MRI data in consensus. They randomly identified, on the pretherapeutic MRI, two hepatic metastases per patient that were larger than 1 cm in size, along with the primary tumor if it hadn’t been previously resected. Inclusion criteria for metastases encompassed a homogeneous appearance and absence of artifacts within the lesion across all sequences. The image review took place in two separate sessions, both achieving consensus: 1) pretherapeutic MRI, and for the sub-analysis 2) post-therapeutic MRI, with a three-week interval between each session.

For quantitative analysis, the size of liver metastases and NETs were measured on the hepatobiliary and arterial phases, respectively. ADCmean and ADCmin values of the tumorous lesions were calculated by manually placing circular regions-of-interest (ROIs) on the slice with the largest tumor extent on DWI, excluding structures near the rim to avoid partial volume effects. Signal intensity (SI) values on non-contrast T1-weighted and T2-weighted images were recorded by outlining ROIs of the lesions as large as possible. Percentage of arterial enhancement was visually assessed by the two radiologists in consensus. Additionally, ADC mean and ADC min values, as well as T2-weighted and T1-weighted SI values of the normal liver, pancreas, and spleen, were measured by placing circular ROIs in tumor-free tissue areas. Additionally, SI of the normal liver was measured on the hepatobiliary phase. Tumor-to-organ ratios, including tumor-to-spleen (T/S) ratio and tumor-to-liver (T/L) ratio of SI and ADC, were calculated.

### Standard of reference and response to treatment

Clinical and surgical records were compiled by a third radiologist. Histopathological confirmed diagnoses of NET, along with their respective Ki-67 indices, were obtained for each patient. Tumor grading adhered to the 2017 WHO Tumor Classification Guideline (G1: Ki-67 Index < 3%, G2: Ki-67 Index 3–20%, and G3 neuroendocrine tumor/neuroendocrine cancer [NET/NEC]: Ki-67 Index > 20%). Given that the primary tumor was resected in 31 out of 44 patients, rendering RECIST 1.1. assessment of treatment response heterogeneous, evaluation of treatment response was conducted through PFS. This was measured in months from the initiation of CAPTEM until progression, as determined by the local interdisciplinary tumor board’s comprehensive assessment of all performed imaging studies (CT, PET/CT, MRI). Responders were defined by PFS ≥ 6 months, while non-responders (NR) were defined by PFS < 6 months, respectively.

**FIGURE 3.**
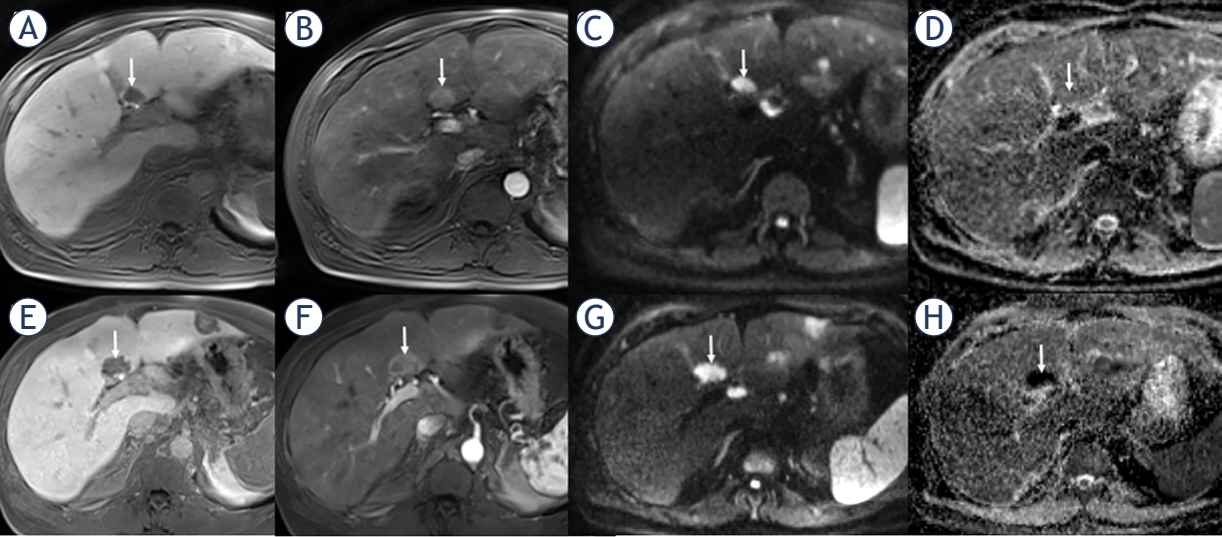
A 56-year-old man with liver metastasis of pancreatic NET classified as nonresponder with a PFS of 3 months. The baseline axial contrast-enhanced T1- weighted image (hepatobiliary phase) **(A)** shows a hypointense lesion (arrow) in segment 4A. The metastasis shows a strong artrerial enhancement **(B)** and restricted diffusion (arrow) with high signal on axial DW-MR image b = 800 s/mm^2^ **(C)** and dark signal (arrow) on ADC map **(D)**. After 3 months under CAPTEM, the metastasis (arrow) **(E)** exhibited an increase in size; however, it shows less arterial enhancement **(F)**. On the axial DW-MR image b = 800 s/mm^2^, the metastasis (arrow) demonstrated hyperintense signal to liver and increasing hypointense signal on the ADC map indicating increasing restricted diffusion compared to the baseline image ADC, apparent diffusion coefficient. ADC = apparent diffusion coefficient; CAPTEM = capecitabine and temozolomide; DW-MR = diffusion-weighted magnetic resonance; NET = neuroendocrine tumor; PFS = progression-free survival

### Statistical analysis

Continuous data were summarized by median with interquartile range (IQR) and categorical data by numbers and percentages. Differences between baseline and follow-up parameters were assessed by Wilcoxon signed-rank test for paired samples. Differences of baseline characteristics and parameter changes until follow-up between non-responder and responder were investigated by Wilcoxon rank-sum test for unpaired samples or Fisher’s exact test. The area under the receiver operating characteristic (ROC) curve (AUC) was estimated according to logistic regression models predicting non-responder by selected imaging and clinical parameters. Two AUC values were compared by chi^2^-test. Sensitivity, specificity, and the Youden-Index were calculated for median-dichotomized parameters. Overall survival (OS) and PFS curves with median survival times were calculated by Kaplan-Meier analysis and compared by log rank-test between individuals separated by the median for selected parameters. Individuals were censored in case of death, progression or end of study. A p-value < 0.05 was considered to indicate statistical significance. All analyses were conducted with Stata 16.1 (Stata Corporation, College Station, TX, U.S.A.).

## Results

### Patients’ characteristics

A total of 44 patients, comprising 86 neuroendocrine liver metastases (NELM) and 14 primary pancreatic NETs were included for the evaluation of prognostic factors for PFS. A subset of 33 patients, with corresponding 66 NELM and 12 pNETs, was identified for the sub-analysis of therapy monitoring. Baseline MRI scans were obtained 19d (IQR 1; 61) prior to CAPTEM initiation, and the time interval between baseline MRI and follow-up MRI was 130 days (IQR 113; 161). Most patients were male (75%), had G2 tumors (76%), and the primary tumor originated in the pancreas (84%). Detailed patient characteristics are presented in Table 1.

**TABLE 1.** Patients characteristics

	**Baseline N = 44**	**Follow-Up N = 33**
Age (years)	60.4 (50.5; 70.2)	
Males	33 (75.0%)	
Time initial diagnosis – therapy start	685 (199; 1230)	
**Clinical parameter**		
Hepatic tumor burden (%)	10 (5 ;40)	
CgA (ng/ml)	610 (119; 2093)	647 (261; 2357)
Bilirubin (mg/dl)	0.6 (0.4; 0.8)	0.7 (0.6; 0.9)
Grading		
1	1 (2.4%)	
2	32 (76.2%)	
3	6 (14.3%)	
NEC = 4	3 (7.1%)	
Ki-67 primary tumor (%)	15 (8;20)	
Localization primary tumor		
Pancreas	37 (84.1%)	
Lung	7 (15.9%)	
**MRI parameter**		
**NELM**		
Size (mm)	28 (19;36)	24.5 (18;38.5)
T1 non-contrast/T1 liver	0.62 (0.53;0.68)	0.68 (0.56;0.75)
T2/T2 liver	1.63 (1.16;2.07)	1.66 (1.21;2.17)
ADCmin	448.5 (242.5;628.5)	549 (341;848)
ADCmean	903 (708.5;1069.5)	969 (764;1250)
ADCmin/ADCmin liver	0.80 (0.60;0.93)	0.85 (0.51;1.32)
ADCmean/ADCmean liver	0.82 (0.74;0.96)	0.99 (0.65;1.32)
% arterial vascularization	42.5 (15;80)	22.5 (5;74.5)^**^
**PNET**		
Size (mm)	43 (32;70)	43 (29.5;52)
T1 non-contrast /T1 pancreas	0.63 (0.59;0.76)	0.68 (0.61;0.84)
T2/T2 pancreas	1.38 (0.85;1.67)	1.08 (0.83;1.34)
ADCmin	604.5 (237;648)	628 (499.5;758.5)
ADCmean	985 (810;1150)	1042.5 (939;1167)
ADCmin/ADCmin pancreas	0.69 (0.41;1.11)	0.73 (0.58;0.85)
ADCmean/ADCmean pancreas	1.01 (0.78;1.19)	0.89 (0.72;0.97)
% arterial vascularization	15 (10;80)	7 (5;45)

Data are given as median (25th and 75th percentile) or number (percentage);

* p < 0.05;

** p < 0.01;

*** p < 0.001 from Wilcoxon signed-rank test;

ADC = apparent diffusion coefficient; CgA = chromogranin A; d = days; NEC = neuroendocrine cancer; NELM = neuroendocrine liver metastasis; PNET = pancreatic neuroendocrine tumor

**TABLE 2.** Differences in baseline clinical and imaging tumor parameters between responder and non-responder

	**Non-responder (< 6 months PFS) N = 23**	**Responder (≥ 6 months PFS) N = 21**	**p-value**
Age	57.8 (44.1;71.1)	61.7 (55.8;68.8)	0.953
Males	16 (69.6%)	17 (81.0%)	0.494
Time ID – Therapy start (d)	851 (426;1552)	396 (153;1004)	0.115
**Clinical parameter**			
Hepatic tumor burden (%)	5 (5;20)	20 (10;40)	**0.007**
CgA	592 (116;2031)	616 (156.5;2745)	0.706
Bilirubin	0.6 (0.4;0.8)	0.6 (0.3;0.9)	0.859
Grading			0.234
1	0 (0%)	1 (5%)	
2	15 (68.2%)	17 (85%)	
3	4 (18.2%)	2 (10%)	
NEC = 4	3 (13.6%)	0 (0%)	
Ki-67 primary tumor (%)	16.5 (10;30)	10.0 (5;15)	**0.013**
Localization primary tumor			0.232
Pancreas	21 (91.3%)	16 (76.2%)	
Lung	2 (8.7%)	5 (23.8%)	
**MRI parameter**			
**NELM**			
Size (mm)	25.5 (17;33.5)	29.8 (21.8;37.5)	0.348
T1 non-contrast/T1 liver	0.60 (0.53;0.68)	0.64 (0.54;0.74)	0.263
T2/T2 liver	1.62 (1.2;2.07)	1.69 (1.12;2.06)	0.903
ADCmin	506 (228;639)	424 (243;606)	0.827
ADCmean	852.5 (674;1059)	911 (790.5;1082.5)	0.495
ADCmin/ADCmin liver	0.80 (0.63;0.93)	0.74 (0.51;1.03)	0.846
ADCmean/ADCmean liver	0.82 (0.68;0.93)	0.86 (0.78;1.02)	0.342
% arterial vascularization	45 (15;85)	36.3 (15;72.5)	0.494
**PNET**			
Size (mm)	38 (30;44)	75.5 (65;85.5)	**0.024**
T1 non-contrast /T1 pancreas	0.60 (0.58;0.71)	0.71 (0.63;0.8)	0.258
T2/T2 pancreas	1.38 (0.84;1.67)	1.38 (1.11;1.5)	0.777
ADCmin	604.5 (237;648)	527 (316.5;698)	1.000
ADCmean	893 (789;1055)	1084 (996.5;1256)	0.157
ADCmin/ADCmin pancreas	0.79 (0.41;1.18)	0.63 (0.44;0.8)	0.480
ADCmean/ADCmean pancreas	1.09 (0.66;1.31)	0.97 (0.96;1.1)	0.888
% arterial vascularization	10 (5;70)	65 (30;85)	0.130

Data are given as median (25th and 75th percentile); p-values are from Wilcoxon rank-sum (Mann-Whitney) test or Fisher’s exact test; ADC = apparent diffusion coefficient; CgA = chromogranin A; NELM = neuroendocrine liver metastasis; PFS = progression-free survival; PNET = pancreatic neuroendocrine tumor

In the baseline cohort, the overall median PFS was 5.7 months (IQR 3.6; 15.0), and median OS was 25.0 months (interquartile range [IQR] 16.3; 45.3). Responder in the baseline group tended to have a slightly longer median OS 35.0 m (IQR 19.4; 53.4) compared to non-responders, with a median OS 21.4 month (IQR 15.0; 38.3). According to RECIST 1.1,21 patients were rated as stable disease (SD), 3 patients were rated as partial response, and 9 patients were graded as progressive disease.

**TABLE 3.** Differences in change of clinical and imaging tumor parameters between responder and non-responder

**Change between baseline and follow-up (%)**	**Non-responder (< 6 months PFS) N = 17**	**Responder (≥ 6 months PFS) N = 16**	**p-value**
**Clinical parameter**			
CgA	61.2 (−8.3;251.9)	−1.5 (−69.3;19)	**0.036**
Bilirubin	0 (−20;40)	8.3 (−15.3;133.3)	0.312
**MRI parameter**			
**NELM**			
Size (mm)	20 (−4.7;50)	−8.0 (−20.1;2.2)	**0.038**
T1 non-contrast/T1 liver	5.4 (−3.8;32.6)	−6.8 (−13.6;11.2)	0.078
T2/T2 liver	1.6 (−9.2;24.1)	−5.7 (−26.2;32.8)	0.589
ADCmin	−22.8 (−41.1;40.2)	49.7 (−6.7;146.4)	**0.037**
ADCmean	−3.5 (−18.4;14.1)	11.7 (−3.4;75.4)	**0.056**
ADCmin/ADCmin liver	−32.3 (−46.2;70.8)	47.5 (12.7;251.7)	0.113
ADCmean/ADCmean liver	−16.3 (−30.6;6.9)	30.0 (6.9;90.4)	**0.011**
% arterial vascularization	−16.7 (−75; −5.9)	−16.7 (−50.0;11.8)	0.298
**PNET**			
Size (mm)	2.3 (−5.4;20)	−55 (−60; −17.8)	**0.013**
T1 non-contrast /T1 pancreas	7.4 (−3.8;36.7)	−5 (−19.7;1.9)	0.116
T2/T2 pancreas	−16.6 (−22;1.2)	−36.1 (−40.3; −10.1)	0.229
ADCmin	14.4 (−13.7;260.8)	18.7 (−33.2;48.9)	0.782
ADCmean	8.3 (−4.5;29.3)	4.0 (−26.3;4.6)	0.405
ADCmin/ADCmin pancreas	−3.6 (−29;76.6)	53 (−18.4;80.9)	0.518
ADCmean/ADCmean pancreas	−23.2 (−35.5;4.5)	−5.7 (−14.3;0.2)	0.518
% arterial vascularization	−50 (−80;0)	−50 (−80;0)	0.851

Data are given as median (25th and 75th percentile); p-values are from Wilcoxon rank-sum (Mann-Whitney) test; ADC = apparent diffusion coefficient; CgA = chromogranin A; NELM = neuroendocrine liver metastasis; PFS = progression-free survival; PNET = pancreatic neuroendocrine tumor

When comparing baseline and follow-up parameters, no differences were observed, except for arterial vascularization of NELM, which was significantly lower at follow-up time.

### Differences between non-responders (NR) and responders (R) at baseline

The comparison of baseline clinical and imaging parameters between the two response groups revealed that NR had a significantly higher Ki-67 of the primary tumor (16.5% *vs*. 10.0%, p = 0.01) with three patients graded as neuroendocrine cancer (NEC) in the NR group (none in the R group). Responders showed a significantly higher hepatic tumor burden (20% *vs*. 5%, p = 0.007). There were no differences in imaging parameters of the NELM, while for the pNETs size varied significantly between response groups with greater diameters of the baseline pNET in R compared to NR (76 mm *vs*. 38 mm, p = 0.02). However, the statistical evaluation of pNET was limited by the small number of patients with non-resected pNET in our cohort (14 and 12 respectively).

### Differences of parameter change between non-responders (NR) and responders (R)

After treatment initiation there was a significant difference in the change of chromogranin A (CgA) between response groups, with an increase in NR compared to a mild decrease in R (61% *vs.* −2%, p < 0.04). Regarding imaging parameters, there were significant differences in the changes of the size of both NELM (20% *vs*. −8%, p = 0.038) and pNET (2% *vs*. −55% p < 0.013) between the two response groups.

Additionally, changes of ADC in NELM differed significantly between response groups, with a decrease in both ADCmin (−23%) and the liver adjusted ADCmean / ADCmean liver ratio (−16%) in NR, compared to an increase in R of both ADCmin (50%) and ADCmean / ADCmean liver (30%). Notably there were no differences in changes in arterial vascularization and signal intensity (SI) on T1w and T2w images between response groups.

### ROC and survival analysis of selected clinical and imaging parameters

**FIGURE 4.**
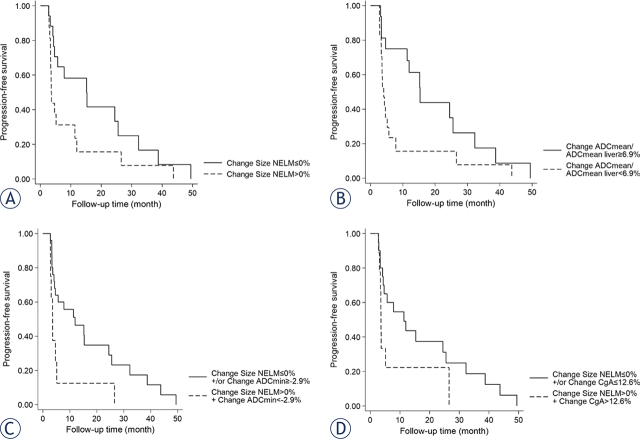
(A) Survival analysis for ∆ size of NELM with a cut-off of ≤ 0% for responder. This cut-off revealed a slightly longer median PFS time of 12.2 *vs*. 3.6 month (p = 0.062). **(B)** The median cut-off for ∆ ADCmean/ADCmean liver showed a significantly longer median PFS time of 15.3 compared to 4.1 month (p = 0.024). Both the combination of ∆ size of NELM > 0% and ∆ ADCmin < − 2.9% and the combination of ∆ size of NELM > 0% and ∆ CgA > 12.6% could differentiate patients with a longer median PFS time. Median PFS of the group with ∆ size of NELM > 0% and ∆ ADCmin < −2.9% was 3.6 m compared to 12 months (p = 0.021) in the group not fulfilling these criteria or a maximum of one criterion. Median PFS of the group with ∆ size of NELM > 0% and ∆ CgA > 12.6% was 3.6 m compared to 11.3 months (p = 0.072) in the group not fulfilling these criteria or a maximum of one criterium. ADC = apparent diffusion coefficient; CgA = chromogranin A; NELM = neuroendocrine liver metastasis; PFS = progression-free survival

ROC analysis of the previously selected imaging and clinical parameters revealed AUC values differing from 0.71 (∆ Size NELM and ∆ ADCmin) to 0.76 (∆ ADC mean/Liver ADCmean) for classifying non-responders *vs*. responders. The highest AUC for a single parameter was found for ∆ ADC mean/Liver ADCmean, with a median cut-off of < 6.9 which yielded a sensitivity of 76% and a specificity of 75%. The combination of ∆ Size NELM and ∆ CgA or ∆ ADC mean/Liver ADCmean could each slightly, though not significantly, improve AUC (0.79 and 0.77 respectively), while the combination of ∆ Size NELM and ∆ ADCmin yielded the best balance for sensitivity and specificity with 88% and 60% compared to 69% and 65% respectively for ∆ Size NELM alone. Subsequent Kaplan-Meier survival analysis, utilizing the respective median cut-off values (Table 4 and Figure 4) for the parameters, revealed significantly longer PFS times for ∆ ADCmean/ADCmean liver < 6.9 (p = 0.024) and the combination of ∆ Size NELM > 0% + ∆ ADCmin < −2.9% (p = 0.021).

## Discussion

In this study, we explored the utility of clinical, morphological, and functional imaging parameters in assessing the response and predicting outcomes in metastatic NETs treated with CAPTEM. Our results underscore the significance of multiparametric MRI, in conjunction with established clinical factors, for evaluating therapy response.

**TABLE 4.** ROC analysis of the previously selected imaging and clinical parameters

	**AUC**	**Cut-off (Median)**	**Sensitivity (%)**	**Specificity (%)**	**Youden-Index**
Ki-67%	0.72	> 15	69	59	0.28
Hepatic tumor burden	0.73	< 10	84	72	0.56
∆ CgA	0.73	> 12.6	67	64	0.31
∆Size NELM	0.71	> 0	**69**	**65**	**0.34**
∆ Size PNET	-	> −2.7	100	50	0.50
∆ ADCmin	0.71	< −2.9	65	63	0.28
∆ ADCmean/ADCmean liver	0.76	< 6.9	76	75	0.51
∆ Size NELM+ ∆ CgA	**0.79**	> 0/> 12.6	78	60	0.38
∆ Size NELM+ ∆ ADCmin	**0.70**	> 0/< −2.9	**88**	**60**	**0.48**
∆ Size NELM+ ∆ ADCmean/ ADCmean liver	**0.77**	> 0/< 6.9	78	58	0.36

All p > 0.05; ADC = apparent diffusion coefficient; AUC = area under the curve; CgA = chromogranin A; NELM = neuroendocrine liver metastasis; PNET = pancreatic neuroendocrine tumor

The median PFS in our baseline cohort was 5.7 months, which is on the lower end of the range of the review by Arrivi *et al.*, which reported a median PFS between 4 to 38.5 months.^6^ Discrepancies may be attributed to the predominance of GEP-NENs (GEP-NENs) in their study. Our median OS aligned well with Arrivi *et al.* report, at 25 months, compared to their range of 8 to 108 months. Disease control rate in our cohort was consistent with the literature, at 73% versus 77%.^6^

Comparison of baseline parameters between non-responders (NR) and responders (R) revealed higher Ki-67 levels (> 15%) in NR, contrasting with some studies suggesting improved response to CAPTEM in tumors with higher Ki-67.^6,12^ The applicability of Ki-67 as a predictive/prognostic biomarker for CAPTEM therapy in NETs remains controversial. Other authors suggested that there was no correlation between tumor grade, mitotic rate, or Ki-67 and tumor response to CAPTEM as the cytotoxic activity of temozolomide is not limited to mitosis but encompasses the entire cell cycle.^7,13^

Responders in our cohort exhibited a higher hepatic tumor burden at baseline, potentially indicating a better response in advanced disease stages. Follow-up analysis revealed marked CgA increases in non-responders versus mild decreases in responders. CgA is considered the most sensitive general marker for the diagnosis of NET^14^, and has been shown to be associated with survival and treatment response^15,16,17,18^ in follow-up, however optimal cut-offs remain controversial.^19^

Changes in size of metastases and primary tumors differed significantly between response groups, and ROC analysis showed an AUC for ∆size NELM of 0.71 with an optimal cut-off of > 0% to define non-response. Generally, we found that cut-offs for tumor progression (≥20%) or response (≥30%) according to RECIST 1.1 were barely reached in our cohort (median ∆size NELM for NR = 20%, and for R = −8%). Therefore, it is critical to adapt treatment response criteria to the rather slow evolution of most NETs to ameliorate management of NET patients and design of clinical trials with better study end points.^19^

An effort to enhance therapy response assessment included the development of mRECIST criteria, initially proposed for hepatocellular carcinoma^20^ and now also proposed an alternative to RECIST for GEP-NETs.^21^ Despite well-developed capillary networks in NETs, and previous indications of DCE-CT perfusion parameters predicting outcomes in NETs undergoing targeted therapies^19,22^, our study revealed a significant decrease in arterial vascularization in both NELM and pNETs after initiating CAPTEM treatment. However, notably, there was no discernible difference between responder and non-responder groups, challenging the utility of mRECIST in this context.

Notably, our investigation revealed significant differences in ADCmin changes and the ratio of ADCmean divided by ADCmean of the liver between response groups. ROC analysis demonstrated the highest AUC for ∆ADCmean/Liver ADCmean, with corresponding cut-offs effectively stratifying patients with longer PFS. Combining changes in tumor size (∆size NELM) with CgA or ADCmin showed slight improvements in sensitivities compared to size-based evaluation alone. Although no study has specifically analyzed the value of ADC for NETs undergoing CAPTEM treatment, existing reports underscore the potential prognostic value of ADC for other treatment strategies.^23,24,25^

Acknowledging study limitations, including its retrospective design and small sample size, future prospective studies with larger cohorts are warranted for validation.

## Conclusions

Our study, among the first to assess multiparametric MRI for monitoring CAPTEM response in hepatic metastasized NETs, suggests the importance of combined evaluation of CgA, ADC values, and tumor size. Our study underscores the complexity of monitoring CAPTEM response in hepatic metastasized NETs, calling for adapted response criteria for slow-growing tumors like NETs, where conventional size-based criteria may not be reached.
